# Charges for Homœopathic Services

**Published:** 1845-02

**Authors:** 


*Charges for Homœopathic Services.*—Perhaps the following report of a suit for the recovery of fees for homœopathic practice, in Cincinnati, Pulte *vs.* Woodruff, may not be unacceptable to physicians generally, since it contains not only the opinions of eminent medical gentlemen of that city on the value of homœopathy, but an expression of the opinion of a jury also. We are indebted for the abstract to the Botanico-Medical Recorder, for which work it was drawn up by a distinguished legal gentleman.

This was a suit to recover a bill of $92, for services rendered the defendant, as a physician.

The defence was that the bill had been settled and paid, and also that the services were of no value.

As to the first point, there was various testimony, pro and con.

As to the second point, it was proved that the plaintiff was employed as a homœopathic physician, and the defendant introduced witnesses for the purpose of proving that medicine administered upon the homœopathic system could not be of any use to the patient. On behalf of the plaintiff, Drs. Peck and Price were examined. Dr. Peck testified that he was educated in the allopathic school, and practised several years, that within some three or four years he had abandoned that system and adopted the homœopathic system; that the two systems did not differ so much in the medicines used, as in the mode and manner of administering them—that in the old system large doses of medicine were given for the purpose of operating upon the bowels, the stomach, or some other vital organ—that in the new mode they gave medicines in such minute quantities that it would not operate upon these organs, but would be incorporated into the system through the nerves and blood, and thus produce its corrective effect. He stated that the homœopathics gave mercury in doses as small as the three hundredth or one thousandth part of a grain. That homœopathics never gave calomel for the purpose of specifically affecting either the bowels or the salivary glands—that they never designed to produce either alvine evacuations or salivation, nor would calomel, administered upon the homœopathic system, ever produce either of those effects—that it was a rule of homœopathic practice never to give but one kind of medicine at the same time, in other words, never to give compound medicines—but their medicines were varied from day to day, as the symptoms varied—that their medicines were mostly given in pills or a powder of the sugar of milk; some, however, were given in liquids—that the homœopathics had two or three hundred different kinds of medicines, which were mostly prepared in Germany, but some in New York. That one material difference, in practice, between the allopaths and homœopaths, was, that the former gave written prescriptions for medicine, which the patient purchased of the apothecary, but the latter always furnished his own, for which he made a distinct charge—that the usual charge was a dollar a visit, and twenty-five cents for medicine.

On being asked if the homœopathic system was not rapidly increasing in the United States, Dr. Peck stated that there were six or seven practitioners upon that system in Cincinnati, sixty or seventy in Philadelphia, about the same number in New York, and about twenty in Boston.

Dr. Price stated that he had been educated in the old school, but within a few years had paid some attention to homœopathy. He stated, however, that he was not, and had not been for some time past, in general practice. His testimony coincided with the testimony of Dr. Peck.

On the part of the defendant, Drs. Wood and Harrison were examined. Dr. Wood stated that he was a practitioner of medicine on the old system—that he was in the habit of giving calomel to his patients, when he thought their cases required it—that it was a very powerful and a very dangerous instrument in unskilful hands; that it required all the skill they were able to acquire to enable them to use it successfully, without injury to the patient—that, from his knowledge of the human system, and of the laws by which it was governed, he did not believe it possible that such minute doses of calomel, as he understood the homœopaths were in the habit of administering, could produce any perceptible effect, nor could he conceive that any medicine administered in such minute doses as the thousandth part of a grain, could affect the system either in sickness or health. The thousandth or three hundredth part of a grain of calomel, taken into the stomach, day by day, for any length of time, would mix with the food and pass off with the fæces, but he could not conceive that it would ever affect the bowels, the salivary glands, the nerves, or any other organ. Dr. Wood had no particular knowledge of the plaintiff or his mode of practice.

Dr. Harrison testified that, being a lecturer and teacher of medicine in the Cincinnati College, he had considered it his duty to make himself acquainted with the homœopathic system of medicine—that he had read the work of Dr. Hahnemann, the original inventor of the system, besides some other works—that in his opinion it was based upon mere hypothesis or the assumption of facts, which never were and never could be proved—that it was contrary to the laws of nature, in regard to the human body, so far as we know them; and that those who believed in it, had no other foundation for their belief than credulity or superstition. That the word homœopathy was derived from two Greek words, one signifying *similar*, and the other signifying *suffering*. That the fundamental maxim of their system was *similia similibus curantur*—like cures like, or whatever will produce disease in a well man, will, if administered in minute doses, cure a sick one. If opium in large doses produces headache and stupor, then opium in small doses will cure that headache and stupor; and if wine or brandy in large doses produces inebriety and vomitings, then wine or brandy in small doses, one drop, or the hundredth part of a drop, will cure that inebriety and vomiting. Dr. Hahnemann states that he made his discovery in consequence of taking a large dose of Peruvian bark when in health, which he found to produce shaking and a headache, and he thence concluded that Peruvian bark given in minute doses would cure the fever and ague. Dr. Harrison further stated that the homœopaths gave medicines for symptoms and not for disease. If they found a patient vomiting, whether the vomiting was caused by inebriety, pregnancy, inflammation, or any other cause, they administered the same medicine. If the patient was suffering a violent pain in the head, the same remedy was applied, whether the pain proceeded from inebriety or inflammation of the brain. He further stated that it was a dogma or first principle of Hahnemann, and of the homœopathic practitioners, that the more a medicine was triturated or diluted, the more powerful it became, and it might be triturated to that degree that the human system could not bear it, but would explode; thus the tenth part of a grain of calomel, when properly triturated and mixed with the sugar of milk, was more powerful than a grain, and the hundredth of a grain more powerful than the tenth, and the thousandth more powerful than the hundredth, and so on indefinitely—that according to the theory of Hahnemann, an ounce of calomel or laudanum put into the Ohio river at Pittsburgh, would, if properly triturated and diffused through the whole body of the river, by the time it arrived at New Orleans, become so powerful as to poison and destroy all who drank of it. This theory Dr. Harrison considered gratuitous and fanciful; yet upon this theory it is, that the homœopaths administer the thousandth part of a grain of calomel. Hence Hahnemann mentions that smelling a medicine is often more efficacious in curing a disease than swallowing it. Dr. Harrison confirmed the testimony of Dr. Wood, that none but experts or proficients should ever administer calomel, especially to women of delicate constitutions. Dr. H. was asked whether there was not a large number of physicians in Cincinnati who practised on the botanical system, and repudiated calomel altogether; but he declined answering the question, as it had nothing to do with this case. Various other testimony was offered respecting the character, education, and qualifications of Dr. Pulte.

Verdict for plaintiff for $27 50.

*Boston Medical and Surgical Journal.*

